# Inflammation – Cause or Consequence of Heart Failure or Both?

**DOI:** 10.1007/s11897-017-0337-9

**Published:** 2017-06-30

**Authors:** Sophie Van Linthout, Carsten Tschöpe

**Affiliations:** 1 Berlin-Brandenburg Center for Regenerative Therapies, Charité – Universitätsmedizin Berlin, Freie Universität Berlin, Humboldt-Universität zu Berlin, and Berlin Institute of Health, Berlin, Germany; 2 Department of Cardiology, Campus Virchow Klinikum, Charité – Universitätsmedizin Berlin, Freie Universität Berlin, Humboldt-Universität zu Berlin, and Berlin Institute of Health, Berlin, Germany

**Keywords:** Heart failure, Sterile inflammation, Para-inflammation, Cardiosplenic axis, Monocytopoiesis, ß-adrenergic signaling

## Abstract

**Purpose of Review:**

With the intention to summarize the currently available evidence on the pathophysiological relevance of inflammation in heart failure, this review addresses the question whether inflammation is a cause or consequence of heart failure, or both.

**Recent Findings:**

This review discusses the diversity (sterile, para-inflammation, chronic inflammation) and sources of inflammation and gives an overview of how inflammation (local versus systemic) can trigger heart failure. On the other hand, the review is outlined how heart failure-associated wall stress and signals released by stressed, malfunctioning, or dead cells (DAMPs: e.g., mitochondrial DNA, ATP, S100A8, matricellular proteins) induce cardiac sterile inflammation and how heart failure provokes inflammation in various peripheral tissues in a direct (inflammatory) and indirect (hemodynamic) manner. The crosstalk between the heart and peripheral organs (bone marrow, spleen, gut, adipose tissue) is outlined and the importance of neurohormonal mechanisms including the renin angiotensin aldosteron system and the ß-adrenergic nervous system in inflammation and heart failure is discussed.

**Summary:**

Inflammation and heart failure are strongly interconnected and mutually reinforce each other. This indicates the difficulty to counteract inflammation and heart failure once this chronic vicious circle has started and points out the need to control the inflammatory process at an early stage avoiding chronic inflammation and heart failure. The diversity of inflammation further addresses the need for a tailored characterization of inflammation enabling differentiation of inflammation and subsequent target-specific strategies. It is expected that the characterization of the systemic and/or cardiac immune profile will be part of precision medicine in the future of cardiology.

## Introduction

Heart failure (HF) is one of the most common disorders in Western societies, and its prevalence is still rising. Approximately 50% of all HF patients suffer from HF with reduced ejection fraction (HFrEF; typically considered as EF <40%), whereas the other half suffers from HF with preserved ejection fraction (HFpEF; ≥EF50). Patients with a left ventricular ejection fraction in the range of 40–49% represent a “gray area,” which is newly defined as HF with mid-range ejection fraction (HFmEF) [1]. The HF epidemic can be explained by the paradox of clinical success, leading to a decrease in mortality due to myocardial infarction (MI) and consequential raise in surviving HF patients, as well as by the increasing prevalence of diabetes mellitus and obesity, which are besides hypertension and COPD, the main comorbidities associated with HFpEF [2].

Our understanding regarding the development and progression of HF has changed over the last decades. Physicians have traditionally considered HF to be a hemodynamic disorder. The inability of the so-called hemodynamic hypothesis to explain the progression of HF and the evidence that activation of the sympathetic nervous system and renin angiotensin aldosteron system (RAAS) exerts a direct deleterious effect on the heart that is independent of the hemodynamic actions of these endogenous mechanisms has then given rise to the notion that HF may progress as a result of the overexpression of neurohormones (neurohormonal hypothesis) [3]. In the 1990s, it has become apparent that in addition to neurohormones, cytokines play an important role in the pathogenesis of HF (cytokine hypothesis) [4]. The last decade, inflammation has more and more been recognized to play an important role in the pathogenesis of both main forms of HF [5, 6•, 7••, 8] and as a consequence, to be an important therapeutic target for the treatment of HF [9, 10]. Common to both forms of HF is the correlation between elevated serum concentrations of pro-inflammatory cytokines and adverse clinical outcomes [11–13]. However, how inflammation contributes to the pathogenesis of both main forms of HF is different. For HFpEF, a novel paradigm was postulated which identifies a systemic pro-inflammatory state induced by comorbidities as the origin of microvascular endothelial cell inflammation, which triggers HFpEF-specific, i.e., concentric, cardiac remodeling, and dysfunction [6•]. With respect to HFrEF, cardiomyocyte damage directly induced by, e.g., myocardial infection or ischemia underlies inflammation triggering eccentric cardiac remodeling and dysfunction. Besides the different causes of inflammation, inflammation itself is diverse and complex, which might explain the disappointing results of anti-inflammatory strategies so far [14, 15]. Indeed, it is more and more recognized that further insights into this diversity and complexity depending on the specific cardiac disorder are required in view of finding target-specific therapies. With the intention to summarize the currently available evidence on the pathophysiological relevance of inflammation in HF, this review addresses the question whether inflammation is a cause or consequence of HF, or both. In general, this question reflects whether inflammation can damage tissue or tissue damage can trigger inflammation, or both. To be able to answer this question, it is first of all necessary to know how inflammation is defined. Therefore, the inflammatory response, sterile inflammation, para-inflammation, and chronic inflammation are briefly discussed. Next, different sources of inflammation and their contribution to HF are outlined, followed by how HF can induce inflammation.

## Inflammation

The first century Roman doctor Cornelius Celsus described the four cardinal signs of inflammation, *rubor et tumor* cum *calore et dolore* (redness and swelling with heat and pain) [16]. Only in 1858, the fifth cardinal sign, *functio laesa* (disturbance of function), was added by Rudolph Virchow [17]. In contrast to the four cardinal signs, which only apply to acute inflammation accompanying wounds and infections, *functio laesa* is the only universal sign of inflammation [16]. A typical inflammatory response consists of four components: (1) the inflammatory inducers, classified in exogenous (microbial inducers including pathogen-associated molecular patterns (PAMPs), virulence factors, and non-microbial inducers: allergens, toxic compounds, irritants) and endogenous inducers (danger-associated molecular patterns (DAMPs): cell-, tissue-, plasma-, extracellular matrix (ECM)-derived products); (2) the sensors that detect them including pattern recognition receptors (PRRs) or other sensors like the nucleotide-binding oligomerization domain-like receptor with a pyrin domain 3 (NLRP3) inflammasome; (3) the inflammatory mediators induced by the sensors (vasoactive amines and peptides, fragments of complement components, lipid mediators, proteolytic enzymes, chemokines, and cytokines); and (4) the target tissues that are affected by the inflammatory mediators [16]. With respect to mediators, this review particularly discusses the relevance of cytokines in HF.

### Sterile Inflammation

If inflammation occurs in the absence of infection, one speaks of sterile inflammation. Post-ischemic or toxic necrosis, massive trauma, hemorrhage, and resuscitation can each trigger an inflammatory response. Molecules released from dying cells, altered host cell products (breakdown products of the ECM, oxidized lipoproteins), and abnormally released host cell products (e.g., heat shock proteins) are involved. Inflammatory responses induced by sterile stimuli are very similar to responses during infection, including the recruitment of neutrophils and macrophages (MΦs), the production of inflammatory cytokines and chemokines, and the induction of T cell-mediated adaptive immune responses [18]. Sterile endogenous stimuli trigger inflammation via (1) activation of PRRs by mechanisms similar to those used by microorganisms and PAMPs, but weaker and delayed as shown for a sterile signal-induced macrophage NLRP3 inflammasome response relative to microbial signals [19]; (2) release of intracellular cytokines which activate common pathways downstream PRRs; and (3) activation of receptors which are not typically associated with microbial recognition like the receptor for advanced glycation endproducts (RAGE) and CD36 [18].

### Para-inflammation

This response is characterized by no massive tissue injury and a limited inflammatory activation. Therefore, it is termed para-inflammation derived from the Greek prefix *παρα/para* for near [20]. This response relies mainly on tissue-resident MΦs. If tissue malfunction is present for a sustained period, para-inflammation can become chronic [20]. This form of inflammation often accompanies obesity, the metabolic syndrome, type 2 diabetes, atherosclerosis, aging, and other chronic inflammatory conditions that are associated with modern human diseases. Para-inflammation is consequently also called “low-grade” chronic inflammation and in case of metabolism-triggered inflammation, “meta-inflammation” [21]. Environmental factors including caloric excess, intake of processed foods, use of antibiotics, and physical inactivity, common to Western lifestyle [22], as well as endocrine disruptors and early life influences (maternal nutrition, placental function) underlie para-inflammation.

### Chronic Inflammation

Chronic inflammation can be caused by persistence of the inflammatory trigger, which disables an appropriate induction of the resolution phase and can occur when there is a positive feedback loop between inflammation and the pathological process it accompanies. Obesity for example can lead to inflammation, whereas chronic inflammation can induce obesity-associated diabetes in part by inducing insulin resistance [21]. With respect to pathophysiological processes in the heart, there is accumulating evidence that inflammation-triggered myofibroblasts are capable of inducing the inflammatory response by their own via (1) expressing chemokines, attracting immune cells to the heart, (2) inducing adhesion molecules on the endothelium, (3) stimulating monocytes to express gelatinases, facilitating transmigration of immune cells through the basolateral membrane [23, 24], and (4) NLRP3 inflammasome activity and IL-1ß release [25]. In this manner, a vicious circle is induced supporting chronic inflammation in the heart [24].

### Inflammation Causes Heart Failure

Inflammation triggers HF in its different aspects, ranging from its impact on the pathogenesis of HF including HF-underlying comorbidities like diabetes and obesity [26, 27], and on pathological substrates underlying heart disease like endothelial dysfunction [28–31] and atherosclerosis [32], to its influence on the progression and outcome of acute coronary syndrome (ACS) [33] and HF [34]. Blood monocyte levels [35] and splenic activity [36] can predict cardiovascular events in patients, C-reactive protein levels are higher in patients with recurrent events [37], and cardiac inflammation is a predictor for a negative outcome in patients with dilated cardiomyopathy [34]. Inflammatory cytokine (TNF-α, IL-1ß, IL-6) levels are increased in HF patients [38]. There is a correlation between serum levels of TNF-α and the severity of the disease [38], and cytokines and cytokine receptors are independent predictors of mortality in patients with advanced HF [39]. The relevance of inflammation in HF follows from experimental studies in animal models of MI, diabetic cardiomyopathy, pressure overload, and myocarditis using knockout [40–42], or transgenic [43] mice, or mice treated with anti-inflammatory or immunomodulatory strategies, including antibodies (e.g., TNF-α antibody [44], IL-6R antibody [45]), inhibitors (IL-1 converting enzyme inhibitor [46]), agonists/antagonists of cytokines/chemokines (IL-2 agonist [47], CCR2 siRNA [48]), statins [49], HDL-raising strategies [29–31, 50, 51], cell therapies including mesenchymal stromal cells (MSC) [52–54], and cardiac-derived stromal cells [55, 56]. The inflammation-induced cardiac pathophysiological mechanisms underlying HF will next shortly be discussed followed by evidence of high-grade and low-grade systemic inflammation affecting HF.

At the latest, since the cytokine hypothesis from the 1990s [4], it is well established that cytokines exert detrimental effects on the heart. Cytokines like TNF-α and IL-1ß downregulate the expression of Ca^2+^-regulating genes including sarcoplasmic reticulum Ca^2+^ ATPase [57] and Ca^2+^-release channel [58], leading to a direct negative inotropic effect as a direct result of alterations in intracellular Ca^2+^ homeostasis in the adult cardiac myocyte [59]. Abnormalities in sarcoplasmic reticulum Ca^2+^ release promote on their turn eccentric myocardial remodeling (eccentric hypertrophy, substantial fibrosis, ventricular dilation) and pump failure, ultimately resulting in overt HF, in response to pressure overload [60]. This points out that inflammation-triggered Ca^2+^ dysbalance can contribute to cardiac remodeling, leading to a vicious circle [61]. TNF-α and IL-1ß further promote cardiomyocyte hypertrophy [62] and the cytokine IL-6 has been demonstrated to increase cardiomyocyte stiffness via reducing the phosphorylation of titin [45]. TNF-α also triggers cardiomyocyte apoptosis [63] and IL-1ß cardiomyocyte pyroptosis [64].

On cardiac fibroblasts, TNF-α and IL-1ß upregulate angiotensin II type 1 receptors (AT1R) and they induce AT1R density in the post-MI heart [65]. The upregulation in AT1 receptor expression enhances the angiotensin (Ang) II-mediated cardiac fibroblast responses that favor fibrosis [66]. TNF-α and IL-1ß neutralization ameliorates Ang II-induced cardiac damage, further supporting synergistic actions of Ang II and TNF-α/IL-1ß [67]. TNF-α also induces TGF-ß [68] and increases the expression of cardiac fibroblast lysyl oxidase (LOX) expression through TGF-β and PI3Kinase signaling pathways [69]. LOX belongs to a family of enzymes [70], including LOX-like 2, responsible for the crosslinking of ECM proteins, including collagen types I and III. The relevance of LOX-like 2 as therapeutic target of cardiac fibrosis and as biomarker for HF has recently been demonstrated [71]. TGF-ß induces fibroblasts to transdifferentiate into active myofibroblasts. Those cells are not only active in producing collagens but they also act as inflammatory support cells via their capacity to express chemokines, to release factors inducing adhesion molecules on endothelial cells, and via their ability to stimulate monocytes to express gelatinases facilitating degradation of the basolateral membrane and subsequent infiltration of immune cells in the heart [23, 24] and their capacity to modulate the MΦ M1/M2 balance [72]. Furthermore, activated fibroblasts promote cardiomyocyte hypertrophy and dysfunction via the release of pro-fibrotic factors, such as TGF-β1, Ang II, and fibroblast growth factor [73, 74].

On (cardiac) endothelial cells, pro-inflammatory cytokines induce adhesion molecule expression [75] and promote subsequent adhesion of immune cells to the endothelium [76] and transendothelial migration [77]. They induce apoptosis in cardiac endothelial cells [78] and oxygen-centered free radicals, which stimulate the elaboration of plasminogen activator inhibitor-1 and collagen by cardiac microvascular endothelial cells. Accordingly, microvascularly mediated inhibition of fibrinolysis may predispose to the persistence of microvascular thrombi, thereby contributing to impaired microcirculatory function, the no-reflow phenomenon, and cardiac dysfunction after ischemia and reperfusion [79]. TGF-ß and Ang II induce endothelial-to-mesenchymal transition, the transition from an endothelial to a fibroblast phenotype [29], a phenomenon, which has been shown to contribute to cardiac fibrosis in a landmark study by Zeisberg et al. [80]. Recently, it has been shown that TNF-α-induced endothelial natriuretic peptide/guanylate cyclase A/cGMP/phosphodiesterase 2 signaling impairs endothelial barrier functions and enhances myocardial inflammatory infiltration in the early phase after an acute infarction [81].

Inflammatory cytokines further promote structural and electrical atrial remodeling via impairment of gap junctions by changes in connexins and via inducing intracellular Ca^2+^-handling abnormalities and atrial fibroblast activation, leading to impaired atrial conduction [82].

### Sources of Inflammation

The cytokines inducing cardiac remodeling and dysfunction can originate from the heart itself (cardiokines) [83], produced by cardiomyocytes [84], cardiac endothelial cells [85], cardiac fibroblasts [25], cardiac tissue MΦs [86], and cardiac infiltrated immune cells, or can be of extra-cardiac tissues including adipose tissue, gut, and lymphoid organs. Failing human myocardium expresses abundant quantities of TNF-α [11]. Cardiomyocytes have TNF-α receptors on their surfaces [87] and these receptors appear to be released into the circulation during HF [11]. The importance of TNF-α in HF has experimentally been shown in transgenic mice where chronic cardiomyocyte overexpression of TNF-α resulted in the development of dilated cardiomyopathy with ventricular hypertrophy, ventricular dilatation, interstitial infiltrates, interstitial fibrosis, rare myocyte apoptosis, diminished ejection fraction, attenuation of β_1_-adrenergic responsiveness, and expression of atrial natriuretic peptide (ANP) in the ventricle [43].

NLRP3 is considered necessary for initiating a profound sterile inflammatory response. Cardiac endothelial cells [85] and cardiac fibroblasts [25] are both important sources of IL-1ß, one of the endproducts of NLRP3 inflammasome activity. By ischemia/reperfusion injury, the NLRP3 inflammasome is activated as indicated by increased NLRP3 expression, caspase-1 activity, and increased IL-1β and IL-18 production. Simulated ischemia/reperfusion-stimulated NLRP3 inflammasome activation in cardiac microvascular endothelial cells, but not in cardiomyocytes [85]. In another study, a marked increase in NLRP3, IL-1ß, and IL-18 mRNA expression was found in the left ventricle after MI, primarily located to myocardial fibroblasts [25]. The relevance of NLRP3 inflammasome activity in HF follows from studies demonstrating that when hearts were isolated from NLRP3-deficient mice, perfused and subjected to global ischemia and reperfusion, a marked improvement of cardiac function and reduction of hypoxic damage was found compared with wild-type hearts [25], whereas Toldo et al. [88] showed that the formation of the inflammasome in acute myocarditis is predictive for the NYHA class and outcome.

In the healthy mouse heart, ≈6 to 8% of non-cardiomyocytes are resident MΦs [86], a number comparable to the frequency of resident MΦs in other tissues. Humans may have comparable numbers, after MI, the MΦ numbers increase in the heart through the combined effects of massive recruitment of circulating monocytes (that become macrophages in tissues) and local self-renewal of tissue-resident MΦs [89]. MΦs are traditionally classified in inflammatory MΦs, often referred to as classical or M1 MΦs, secreting pro-inflammatory cytokines as IL-6, TNF-α, IL-1β, IL-12, and IL-23, and heal/growth-promoting MΦs, commonly called alternatively activated or M2 MΦs, expressing anti-inflammatory IL-10 and TGF-ß [90]. M1 and M2 MΦs usually appear in sequence upon MI, i.e., in the inflammatory versus the wound healing phase, respectively, whereas also mixed M1/M2 phenotypes can be found [91]. The relevance of cardiac M1 toward M2 MΦ phenotype transition for the resolution of inflammation and tissue repair post MI has recently been shown by Courties et al. (2014) [92] who demonstrated that in vivo silencing of the transcription factor IRF5, which is involved in inflammatory M1 MΦ polarization, supported resolution of inflammation, accelerated infarct healing, and attenuated development of post-MI HF.

Besides endogenous cardiac cells, infiltrated inflammatory cells are responsible for local cardiac cytokine expression. Those immune cells originate from lymphoid organs as the spleen and the bone marrow [93]. Pre-clinical studies have demonstrated that after MI in mice, monocyte progenitor cells depart bone marrow niches, which results in amplified extramedullary monocytopoiesis [36, 94]. The observation of the activation of splenic monocytes and the migration of pro-inflammatory monocytes from the spleen to the heart in animal models of MI [95] and chronic HF [96] have given rise to the concept of a cardiosplenic axis. This recruitment from the spleen depends in part on Ang II, an observation that may underlie the beneficial effect upon angiotensin converting enzyme inhibition on remodeling in the infarcted myocardium [97]. In accordance with the cardiosplenic axis and the immunomodulatory properties of MSC [52, 53], we recently demonstrated that intravenous MSC application in CVB3-induced myocarditis modulates monocytes trafficking to the heart. They reduced blood and cardiac pro-inflammatory monocytes and retained those in the spleen, whereas MSC increased anti-inflammatory monocytes in the spleen, blood, and heart [54].

Evidence for the existence of a cardiosplenic axis is further supported by observations in human post-mortem tissue specimens of the heart, spleen, and bone marrow demonstrating a unique spatio-temporal pattern of monocyte accumulation in the human myocardium following acute MI that coincides with a marked depletion of monocytes from the spleen, suggesting that the human spleen contains an important reservoir function for monocytes [98]. Patients with acute MI exhibit an increased inflammatory status/metabolic activity of the spleen, bone marrow, and carotid artery. This has been demonstrated via ^18^F-fluorodeoxyglucose (^18^F-FDG) positron emission tomography, which evaluates the metabolic activity based on the finding that activated inflammatory cells express high levels of glucose transporters and accumulate ^18^F-FDG [93]. Emami et al. [36] further demonstrated that after ACS, the gene expression of circulating pro-inflammatory monocytes (i.e., CD36, S100A9, IL-1ß, and TLR4) was more closely associated with the metabolic activity of the spleen than it was for the bone marrow. They further observed that the metabolic activity of the spleen independently predicted the risk of subsequent cardiovascular disease events. In patients with acute MI, high monocyte blood levels, which are a strong predictor of mortality, correlate inversely with the ejection fraction [99]. Collectively, the abovementioned findings provide evidence of a cardiosplenic axis in humans similar to that shown in pre-clinical studies [36].

### High-Grade Systemic Inflammation

Evidence from chronic immune-mediated diseases like rheumatoid arthritis associated with persistent high-grade systemic inflammation demonstrates the impact of systemic inflammation on HF. Patients with rheumatoid arthritis have a 1.5–2.0 times higher prevalence of ischemic heart disease and congestive HF compared to the general population [100]. Furthermore, atherosclerosis progresses most rapidly during the first 6 years after rheumatoid arthritis diagnosis [101], indicating how enduring systemic inflammation plays a major role in accelerating heart disease development in these patients. Systemic inflammation can induce autonomic nervous system dysfunction. Inflammatory cytokines increase the sympathetic outflow by targeting the autonomic centers in the brain, which in turn inhibits cytokine production and immune-inflammatory activation by stimulating the ß2 adrenoreceptors in circulating lympho-monocytes [102]. This self-controlling loop, so-called inflammatory reflex [103], and in this context, sympathetic activation, consequently damps excessive immune-inflammatory activation, but also affects the heart, potentially favoring the onset of arrhythmias [104] and HF. In extreme cases of inflammation as systemic inflammatory response syndrome (SIRS), or sepsis, the hemodynamic changes due to hypotension may directly underlie the induction of the neuroendocrine system, independent of the inflammatory response.

### Low-Grade Systemic Inflammation

Obesity is characterized by a low-grade systemic chronic inflammatory state [26]. The multisystem effects of obesity are linked to an imbalance in homeostatic and pro-inflammatory immune responses. A major player in systemic low-grade chronic inflammation in obesity is the increased numbers of adipose tissue pro-inflammatory MΦs and deregulated production and function of adipose tissue hormones and adipokines including adiponectin [105], which strongly contributes to the initiation and exacerbation of type 2 diabetes [106]. Over time, ectopic lipid accumulation in the muscle, liver, and blood vessels activates tissue leukocytes, contributes to organ-specific disease, and exacerbates systemic insulin resistance. Cellular- and cytokine-mediated inflammation in the pancreatic islets accelerates the progression toward diabetes [26]. The obesity-associated alterations in adipokine expression (adiponectin ↓, TNF-α ↑) also contribute to HFpEF [106]. Indeed, adiponectin deficiency known to exacerbate the development of obesity-related hypertension [107], adverse cardiac remodeling [108] in ischemia-reperfusion injury [109], and MI [110], increased the propensity to develop diastolic HF and diastolic dysfunction in a murine model of HFpEF/diastolic HF [111]. In contrast, adiponectin overexpression in aldosterone-infused mice ameliorated left ventricular (LV) hypertrophy, diastolic dysfunction, lung congestion, and myocardial oxidative stress without affecting the blood pressure and LV ejection fraction [112].

Diabetes and obesity both induce hematopoiesis and myelopoiesis. Hyperglycemia promotes myelopoiesis via interaction of neutrophil-derived S100A8/A9 with RAGE on hematopoietic stem cells [113]. S100A8/A9-induced TLR4/MyD88 and NLRP3 inflammasome-dependent IL-1ß production in adipose tissue MΦs interacts with the IL-1 receptor on bone marrow myeloid progenitors to stimulate the production of monocytes and neutrophils. These studies uncover a positive feedback loop between adipose tissue MΦs and bone marrow myeloid progenitors and suggest that inhibition of TLR4 ligands or the NLRP3-IL-1ß signaling axis could reduce adipose tissue inflammation and insulin resistance in obesity [114].

In line with the HFpEF paradigm postulated by Paulus and Tschöpe [6•], it has been demonstrated that the systemic, low-grade inflammation of metabolic risk contributes to diastolic LV dysfunction and HFpEF through coronary microvascular endothelial activation, which alters paracrine signaling to cardiomyocytes and predisposes them to hypertrophy and high diastolic stiffness [115, 116]. In detail, the authors showed upregulated E-selectin and intercellular adhesion molecule-1 expression levels, increased NADPH oxidase (NOX) 2 expression in MΦs and endothelial cells but not in cardiomyocytes, and uncoupling of endothelial nitric oxide synthase, which was associated with reduced myocardial nitrite/nitrate concentration, cGMP content, and protein kinase G activity in the myocardium of HFpEF patients and ZSF1-HFpEF rats. The ZSF1-HFpEF rats are characterized by titin hypophosphorylation and cardiomyocyte stiffness and do not exhibit cardiac fibrosis [116], the other main contributor to cardiac diastolic dysfunction [5, 117, 118]. Murdoch and coworkers [119] demonstrated how Ang II-induced endothelial NOX 2 activation had profound pro-fibrotic effects in the heart in vivo that lead to a diastolic dysfunction phenotype. Endothelial NOX 2 had pro-inflammatory effects and enhanced endothelial-to-mesenchymal transition, which might be an important mechanism underlying cardiac fibrosis and diastolic dysfunction during increased renin-angiotensin activation. A positive correlation between cardiac collagen, the amount of inflammatory cells, and diastolic dysfunction evident in HFpEF patients further suggests a direct influence of inflammation on fibrosis contributing to diastolic dysfunction [5].

Many studies have indicated that an overactive RAAS, excess oxidative stress, and excess inflammation in the brain cause sympathoexcitation in HF [120]. Partial silencing of brain TLR4 via intracerebroventricular injection of TLR4 siRNA causes sympathoinhibition with the prevention of left ventricular remodeling in MI-induced HF through the reduction of brain pro-inflammatory cytokines [120]. Kishi [121] recently demonstrated that systemic infusion of Ang II directly affects brain AT1R with sympathoexcitation and LV diastolic dysfunction. Furthermore, they demonstrated that targeted deletion of AT1R in astrocytes strikingly improved survival with prevention of LV remodeling and sympathoinhibition in MI-induced HF. Based on these results, the authors propose a novel concept that the brain works as a central processing unit integrating neural and hormonal input, and that the disruption of dynamic circulatory homeostasis mediated by the brain causes HF.

## Heart Failure Causes Inflammation

**Fig. 1:**
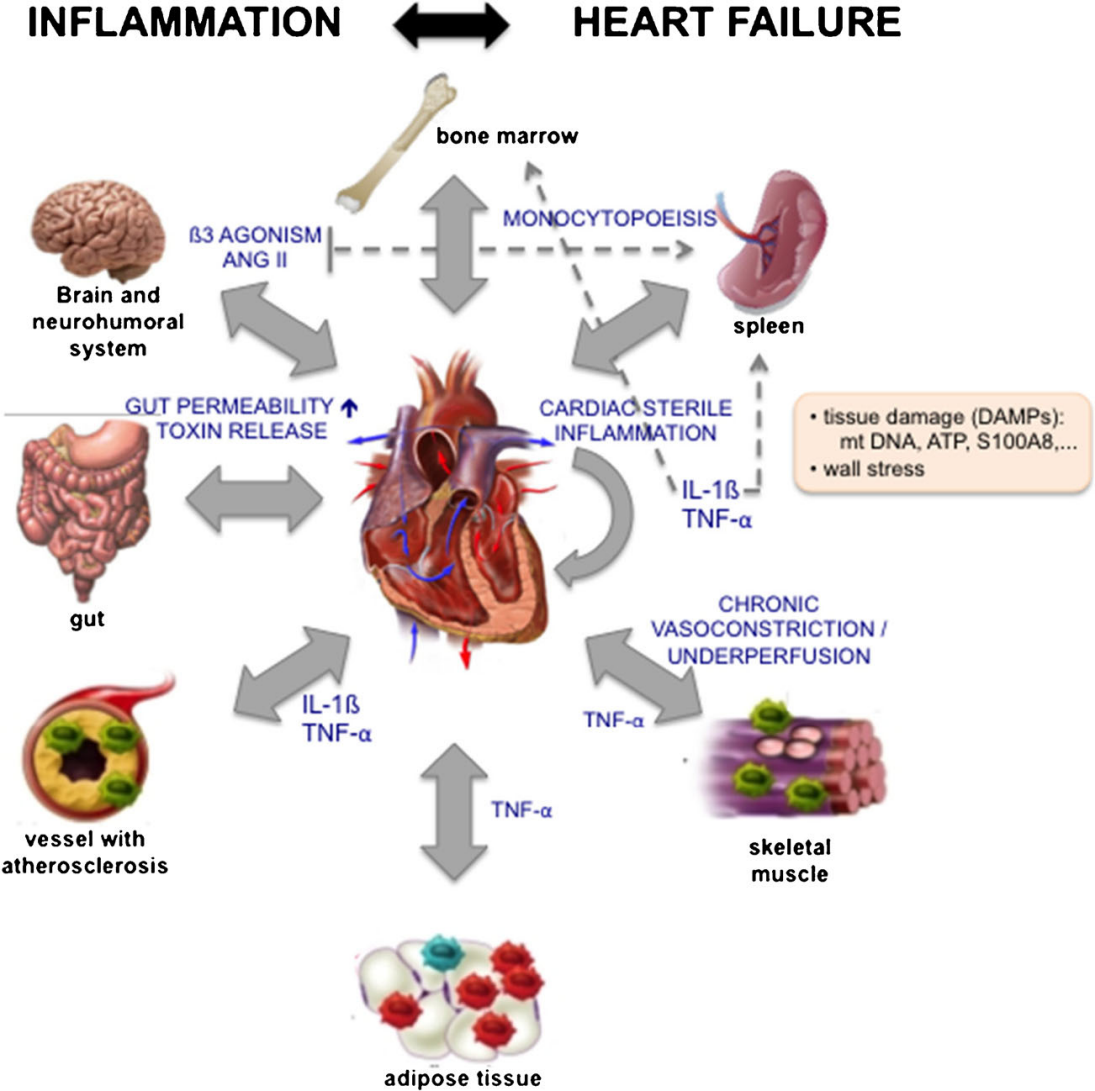
Inflammation and heart failure reciprocally trigger each other. Heart failure (HF) provokes sterile inflammation in the heart itself triggered by wall stress and signals released by stressed, malfunctioning, or dead cells secondary to HF (DAMPs: e.g., mitochondrial (mt) DNA, ATP, matricellular proteins). The released cardiac cytokines and other inflammatory mediators not only affect the heart but also different organs. IL-1ß induces monocytopoiesis via increasing hematopoietic stem cell proliferation in the bone marrow and monocyte proliferation in the spleen. Cytokines particularly, TNF-α, unleashes inflammation in the skeletal muscle and adipose tissue and accelerate atherogenesis. Furthermore, several neurohormonal mechanisms (renin angiotensin aldosteron system (RAAS) and ß-adrenergic nervous system) that become activated in HF to try and sustain cardiac output in the face of decompensating function also affect inflammation in different organs. ß3 agonism and Ang II induce monocytopoiesis in the spleen. As a consequence of chronic vasoconstriction and underperfusion, inflammation is induced in the skeletal muscle. HF-associated decreased cardiac output and redistribution of systemic circulation can further also lead to a decrease in intestinal perfusion and mucosal ischemia and ultimately, a disrupted intestinal mucosa. This disruption can in turn lead to increased gut permeability and subsequent enhanced translocation of bacteria and bacterial toxins in the blood, which can contribute to systemic inflammation. Systemic inflammation, high-grade (e.g., rheumatoid arthritis) and low-grade (e.g., obesity), and cardiac inflammation induce HF involving different pathophysiological mechanisms. Inflammation triggers cardiomyocyte apoptosis, hypertrophy, stiffness, myofibroblast differentiation, collagen production, endothelial dysfunction, endothelial-to-mesenchymal transition, and subsequent cardiac remodeling and left ventricular dysfunction

HF is a clinical diagnosis secondary to either LV systolic or diastolic dysfunction leading to insufficient oxygen and nutrient supply to peripheral organs. HF may underlie different etiologies ranging from ischemic heart disease, valve dysfunction, hypertension, metabolic syndrome, and genetic cardiomyopathies to inflammatory cardiomyopathy. HF induces sterile inflammation in the heart itself triggered by wall stress and signals released by stressed, malfunctioning, or dead cells secondary to HF and induces inflammation in various peripheral tissues in a direct (inflammatory) [122] and indirect (hemodynamic) manner (Fig. 1). Cardiac cells release regulatory peptides, cardiokines, in response to changes in the cardiac environment. These cardiokines affect the heart (see supra) and also have physiological and pathological roles in organs distal from the heart, such as the spleen, bone marrow, adipose tissue, and muscle, affecting cell death, growth, fibrosis, remodeling, metabolism, and inflammation [122, 123].

### Heart Failure Provokes Cardiac Inflammation

Wall stress increases in the failing heart, exposing all cells to increased biomechanical strain. Mechano-sensitive adhesion proteins, including integrins and cadherins, transduce mechanical signals between cells and their microenvironment and can stimulate cellular responses including cell growth, differentiation, and inflammation [124]. TNF-α, IL-6, IL-18, and ANP can be induced in stretched myocytes and in hemodynamic-overloaded myocardium [125]. Cyclic stretch enhances the expression of TLR4 in cultured cardiomyocytes via p38 MAP kinase [126], which is known to mediate inflammatory cytokine induction in cardiomyocytes. Cardiac fibroblasts are activated by mechanical stretch mimicking cardiac dilation in heart failure. Upon stretching, they not only produce more ECM but also upregulate chemokine production and trigger typical inflammatory pathways. Cell culture supernatant of stretched fibroblasts activates inflammatory cells and induces further recruitment of monocytes by allowing transendothelial migration into the cardiac tissue [23]. Furthermore, the mechanical stretch of cardiac fibroblasts, rather than of cardiomyocytes, leads to the release of IL-1ß [127], which induces leukopoiesis in the bone marrow and at the extramedullary sites [128].

Wall shear stress on endothelial cells plays an important role in blood vessel physiology and pathology. In regions where undisturbed wall shear stress dominates, endothelial cells are healthy, whereas regions with disturbed wall shear stress have endothelial cells with a pro-inflammatory, pro-oxidative stress phenotype and represent sites where atherosclerosis preferentially develops [129]. Despite substantial evidence for the central role of hemodynamic shear stress in the functional integrity of vascular endothelial cells, hemodynamic and molecular regulation of the endocardial endothelium lining the heart chambers remains understudied [130]. A recent study by McCormick et al. [130] demonstrated spatio-temporal wall shear stress values in defined regions of the left ventricle linking local hemodynamics to regional heterogeneity in endocardial gene expression. However, the spatial regulation of inflammation in cardiac endothelial cells in response to shear stress requires further investigation. MΦs respond to strain by inflammatory activation, including increased expression of TNF-α, IL-8, IL-6, and MMP-9 [131] and increased expression of scavenger receptors [132]. These phenomena could be of importance in hypertension, which exposes arterial MΦs to increased mechanical forces [132]. A recent study of Sager et al. [89] demonstrated that the mechanical strain of primary murine and human MΦ cultures promoted a cell cycle entry, suggesting that the increased wall tension in post-MI HF stimulates local MΦ proliferation.

Besides increases in wall stress, HF is associated with cell death, oxidative stress (ROS), hypoxia, and ECM remodeling. TLRs and RAGE are important PRRs for the recognition of endogenous DAMPS including the intracellular S100 proteins, heat shock protein, HMGB1, matricellular proteins, and mitochondrial DNA, released by the heart during HF. Stimulation of TLRs in cardiomyocytes initiates a NF-kB-dependent inflammatory response [133]. Extracellular mitochondrial DNA activates NF-kB via TLR9 in cardiomyocytes [134], and heat shock protein 60 induces inflammation through activating and upregulating TLRs in cardiomyocytes [135]. The alarmin S100A1 is released from ischemic cardiomyocytes and signals myocardial damage via TLR4 [136], whereas the alarmin S100A8/A9 aggravates post-ischemic HF through activation of RAGE-dependent NF-κB signaling [137]. An HMGB1-TLR4 axis is active upon myocardial ischemia/reperfusion injury and the innate immune adaptor MyD88 downstream TLR4 has been shown to mediate neutrophil recruitment and myocardial injury after ischemia-reperfusion in mice. [138]. Matricellular proteins such as tenascin-C and the small leucine-rich proteoglycan biglycan modulate the inflammatory response by binding to TLR2 and/or TLR4 [139, 140]. The matrix component biglycan activates the NLRP3 inflammasome via TLRs and P2X receptors and leads to subsequent IL-1ß release [141]. Other important factors by which HF increases cardiac NLRP3 inflammasome activity are ATP, released when cells die [142], ROS, and mitochondrial DNA. ROS mediates autocrine and paracrine activation and nuclear translocation of NF-κB, which regulates the transcription of pro-IL-1ß and pro-IL-18 [143•]. Mitochondrial DNA released by damaged cells [144] directly primes NLRP3 and ATP via binding to P2X7 purinergic receptors and leads to potassium efflux, which triggers the assembly of NLRP3 inflammasome. These collective effects result in the activation of NLRP3-associated caspase 1, which processes pro-IL-1ß and pro-IL-18 in their mature IL-1ß and IL-18 forms [122]. The abovementioned released cardiac cytokines and other inflammatory mediators not only affect the heart but also different organs as outlined below. Furthermore, several neurohormonal mechanisms that become activated in HF to try and sustain cardiac output in the face of decompensating function [145, 146] also affect inflammation in different organs.

### Heart Failure Induces Monocytopoiesis in the Bone Marrow and Spleen

Monocytes arise from hematopoietic stem cells in the bone marrow, pass through several intermediate progenitor stages (from granulocyte MΦ progenitor to MΦ dendritic cell progenitor) [147], and emigrate from the bone marrow into the blood pool mediated by the chemokine receptor CCR2 [148]. IL-1ß released upon MI induces leukopoiesis in the bone marrow and at extramedullary sites [128, 149]. The relevance of IL-1ß in this process follows from MI mice lacking the IL-1R which exhibits an impaired splenic monocytopoiesis as indicated by a reduced number of colony-forming units, less MΦ dendritic cell progenitors, and proliferating monocytes in the spleen [149]. The reduced CD45.2+ progeny in the spleen following adoptive transfer of IL-1R^−/−^ compared to wild-type granulocyte MΦ progenitors indicates that direct IL-1 signaling on myeloid progenitors controls splenic monocytopoiesis. Sager et al. [128] further demonstrated that anti-IL-1ß treatment dampens the post MI increase in hematopoietic stem cell proliferation in the bone marrow. Finally, HF-associated activation of the RAAS also boosts the release of monocytes from their splenic reservoir [97]. The increased monocytopoiesis post MI accelerates coronary plaque growth after the first MI [150] and may be responsible for the high secondary event rates [151].

### Heart Failure Provokes Inflammation in the Skeletal Muscle

It is well established that HF is associated with skeletal muscle wasting and cachexia including increased degradation of myofibrils, myocyte apoptosis, and metabolic imbalance [152]. Less known is the evidence from experimental [153] and patient [154] studies which indicate that HF-elevated serum cytokine levels (most notably TNF-α) are associated with increased local inflammation in the skeletal muscle. The TNF-α/IL-10 and IL-6/IL-10 ratio is higher in the soleus muscle of rats with HF compared to that of controls [155]. The skeletal muscle of patients with chronic HF with only mildly elevated serum cytokines exhibits increased expression of TNF-α, IL-1ß, IL-6, and iNOS compared to skeletal muscle of the control patients [154]. This occurs in the absence of infiltrating monocytes or MΦs indicating that skeletal myocytes may produce cytokines in a paracrine/autocrine fashion [156]. Furthermore, HF-associated inflammation induces resistance to the anti-inflammatory adipokine adiponectin in the muscle [157]. Interestingly, a recent study demonstrated an increase in markers of muscle atrophy, oxidative stress, and mitochondrial impairments solely in the soleus muscle of HFrEF, but not of HFpEF rats. The authors concluded that this disparity may be mediated, in part, by the different circulating inflammatory cytokines that were elevated between HFpEF and HFrEF, i.e., TNF-α plasma concentrations were significantly increased in HFrEF, whereas IL-1β and IL-12 were higher in HFpEF rats [158]. Unfortunately, the analysis of potential differences in skeletal muscle inflammation between HFpEF and HFrEF rats was beyond the scope of this study. Besides inflammation, HF-associated neurohumoral activation triggers inflammation in the skeletal muscle. Chronic sympathetic stimulation in HF promotes redistribution of blood flow to skeletal muscles through chronic vasoconstriction [152]. Chronic underperfusion of the capillary bed, in turn, promotes skeletal muscle ischemia, which leads to the generation of ROS and muscle inflammation [159]. Furthermore, increased levels of Ang II associated with HF result in impaired vasodilation and aggravate bradykinin degradation, leading to muscle hypoxia and reduced endurance capacity [160]. Gielen et al. [154] described that a 6-month program of regular physical exercise significantly reduced the local expression of TNF-α, IL-1ß, IL-6, iNOS, and nitrotyrosine levels in the skeletal muscle of patients with stable moderate chronic systolic HF, while serum cytokine levels remained virtually unchanged. The lower total peripheral resistance after training [161] may contribute to lower oxidative stress and inflammation in the skeletal muscle.

### Heart Failure Affects Inflammation in Adipose Tissue

Pro-inflammatory cytokines are known to reduce the expression of the anti-inflammatory adipokine adiponectin in cultured adipocytes [162] and adiponectin expression in adipose tissue and circulating adiponectin levels are decreased in experimental severe inflammation [163]. However, in humans, adiponectin regulation is complex. Atherosclerosis-related low-grade inflammation has been associated with decreased plasma adiponectin [164], whereas advanced, chronic inflammation with increased adiponectin levels [165]. Antonopoulos et al. [166] demonstrated how the reciprocal effects of systemic inflammation and brain natriuretic peptide (BNP) influence adiponectin expression in patients with HF. Low-grade inflammation reduces the adiponectin levels in populations without significant cardiovascular disease and low plasma BNP, explaining why its low levels predict the onset of cardiovascular disease [167]. However, after the development of advanced cardiovascular disease, the adiponectin levels are no longer negatively controlled by low-grade inflammation, but they are driven upward by circulating BNP levels [168]. Therefore, high circulating adiponectin predicts (indirectly) worse clinical outcome in patients with HF. Therefore, the interpretation of adiponectin as a biomarker should always take into account the underlying cardiovascular disease state [166]. Valero-Munoz et al. [169] recently demonstrated increased neutrophil content in white adipose tissue of HFpEF patients compared to controls. Neutrophils are recognized as primary effector cells in acute inflammatory responses and are implicated in the modulation of adipose tissue inflammation in the early stages of obesity, but their presence in adipose tissue in response to a high-fat diet may last ≤90 days [170]. Therefore, the increased neutrophil presence in white adipose tissue of HFpEF patients suggests the onset of immune activation, setting the stage for tissue infiltration by other immune cells, such as MΦs [169].

### Heart Failure Increases Gut Permeability and Subsequent Systemic Inflammation

HF-associated decreased cardiac output, elevated systemic congestion, and distribution of systemic circulation can lead to a decrease in intestinal perfusion and mucosal ischemia and ultimately a disrupted intestinal mucosa. This disruption can in turn lead to increased gut permeability and subsequent enhanced translocation of bacteria and bacterial toxins in the blood, which can contribute to systemic inflammation and further to HF exacerbations [171]. The HF-associated gut luminal hypoxia and decrease in mucosal pH [172], well known activators of bacterial virulence in microbiota, can also change the microbiota to pathogenic microbiota which further contributes to the raise in gut permeability. A recent study in fact demonstrated that patients with chronic HF have intestinal overgrowth of pathogenic bacteria and *Candida* species and showed that the increased intestinal permeability correlated with systemic inflammation [173]. Beyond their effect on systemic inflammation, the low-level leakage of bacterial products could augment local inflammation of the plaque in vessels, promoting atherogenesis. This is an example of how sites of tissue injury and ischemic damage beyond the myocardium can elicit an “echo” at the level of the atherosclerotic plaque and induce a remote inflammatory response [7••]. This further explains how patients with a primary MI have a higher prevalence to get recurrent ACSs [151].

### Heart Failure Affects Neurohumoral System-Dependent Inflammation and Monocytopoiesis

Pro- and anti-inflammatory cytokine production is regulated by the adrenergic nervous system. Previous studies have demonstrated that β2-, but not β1-receptor agonists attenuate TNF-α expression, while increasing anti-inflammatory IL-10 production [174]. Conversely, α1,2-adrenergic stimulation results in increased expression of TNF-α and reduction in IL-10 [175]. Under normal physiologic conditions, norepinephrine, an α- and β-agonist, reduces TNF-α and enhances IL-10 expression in monocytes exposed to LPS and other stimuli [176]. However, in HF, a paradox exists as both catecholamines and TNF-α are elevated [174]. Ngu et al. [174] recently demonstrated that the norepinephrine regulation of monocyte inflammatory cytokine balance is impaired in HF. The inhibitory effect of noradrenaline on TNF-α production of monocytes from HF patients was lower compared to that of monocytes from control patients, whereas the increase in IL-10 production by noradrenaline was also attenuated in HF monocytes. Another example how HF affects the adrenergic nervous system and inflammation is the ß3-adrenergic-stimulated activation of bone marrow progenitor cells following MI. The pain and acute stress of the acute MI promotes local catecholamine synthesis in the bone marrow and the systemic release of ß3-adrenergic stimulants [7••, 94] The relevance of adrenergic signaling in monocytopoiesis in ischemic disease follows from findings showing that patients undergoing ACS who were non-randomly allocated to ß-blocker use before the ACS had significantly lower leukocyte and monocyte counts than those who had never used ß-blockers [7••, 94]. Finally, HF activates the RAAS, which also triggers monocytopoiesis as outlined before [97].

## Conclusions and Perspectives

Inflammation and HF are strongly interconnected and mutually reinforce each other. This indicates the difficulty to counteract inflammation and HF once this chronic vicious circle has started and points out the need to control the inflammatory process at an early stage avoiding chronic inflammation and HF. The relevance of the ß-adrenergic system in HF as well as in the control of inflammation (inflammatory reflex versus ß3 agonism-induced monocytopoiesis) warrants further investigation. The diversity of inflammation further addresses the need for a tailored characterization of inflammation enabling differentiation of inflammation and subsequent target-specific strategies. This necessity is supported by the disappointing results of anti-inflammatory strategies used in HF patients so far [14, 15]. The characterization and differentiation of inflammation will allow classification of patients in subclasses to provide appropriate treatment. Such a differentiated approach is in line with the growing appreciation and ongoing introduction of precision medicine in cardiology [177], a field of medicine which is a common practice in oncology [178]. It is expected that the characterization of the systemic and/or cardiac immune profile will be part of precision medicine in the future of cardiology. The questions at this stage rise how long this will still take, which methods will be used to that end, and which novel therapeutic targets will be defined.

## Abbreviations

ACS: Acute coronary syndrome
Ang II: Angiotensin II
ANP: Atrial natriuretic peptide
AT1R: Angiotensin II type 1 receptor
BNP: Brain natriuretic peptide
DAMP: Danger-associated molecular pattern
ECM: Extracellular matrix
HF: Heart failure
HFmEF: Heart failure with mid-range ejection fraction
HFpEF: Heart failure with preserved ejection fraction
HFrEF: Heart failure with reduced ejection fraction
LOX: Lysyloxidase
LPS: Lipopolysaccharide
LV: Left ventricular
MΦ: Macrophage
MI: Myocardial infarction
MSC: Mesenchymal stromal cell
NADPH: Nicotinamide adenine dinucleotide phosphate
NLRP3: Nucleotide-binding oligomerization domain-like receptor with a pyrin domain 3
NOX: NADPH oxidase
PAMP: Pathogen-associated molecular pattern
PRR: Pattern recognition receptors
RAGE: Receptor for advanced glycation endproducts
RAAS: Renin angiotensin aldosteron system
ROS: Reactive oxygen species
SIRS: Systemic inflammatory response syndrome
TLR: Toll-like receptor
TNF: Tumor necrosis factor
TGF: Transforming growth factor
